# Maternal smoking during pregnancy and health outcomes in offspring

**DOI:** 10.1186/s12916-022-02507-w

**Published:** 2022-08-08

**Authors:** Hongjiang Wu, Andrea O. Y. Luk

**Affiliations:** Department of Medicine and Therapeutics, The Chinese University of Hong Kong, Hong Kong, Special Administrative Regional People’s Republic of China

**Keywords:** Maternal smoking, Type 1 diabetes, Perinatal epidemiology

## Background

Maternal smoking during pregnancy (MSDP) is an important public health issue that adversely impacts health outcomes of both children and mothers, contributing to low birthweight, preterm birth, miscarriage, and ectopic pregnancy [1]. Despite increasing public awareness on the detrimental effects of MSDP, the proportion of women who smoke during pregnancy is still high in some countries. The global prevalence of MSDP was estimated to be 1.7% in 2015, and it varied substantially across regions, being the most prevalent in Europe (8.1%) and Americas (5.9%) and the least prevalent in Africa (0.8%) and Eastern Mediterranean (0.9%) [2].

## Maternal smoking and risk of type 1 diabetes in the offspring

Type 1 diabetes represents less than 5% of the total cases of diabetes globally, but it is one of the most common chronic diseases of childhood [3]. In contrast to many other health outcomes, several large cohort studies suggested that exposure to maternal smoking may decrease the risk of type 1 diabetes in the offspring [4–6], although this was not confirmed by all studies [7]. However, observational studies are limited by issues of potential confounding. The observed inverse association between MSDP and offspring risk of type 1 diabetes in cohort studies is prone to bias due to unmeasured confounders, such as shared genetic and early life environmental factors within families. These factors are frequently correlated with MSDP to influence offspring health outcomes.

To control for potential confounders from unmeasured familial background factors, apart from a traditional cohort study design, Wei et al. [8] used a family-based, nested case-control study by matching children with type 1 diabetes to their siblings and cousins who were free of diabetes. Sibling and cousin comparisons are quasi-experimental designs, which use design features to help minimise confounding effects from genetic and environmental factors that are shared by family members [9]. The study by Wei et al. included about three million children born in Sweden between 1983 and 2014 and followed them until 2020. The overall proportion of children exposed to MSDP in this study population was high, with a prevalence of 15.7%. The study found that children exposed to MSDP had a 22% (95% CI: 18%, 25%) reduced risk of developing type 1 diabetes during childhood compared to their unexposed siblings. Similar association was observed in cousin comparison analysis (odds ratio: 0.72, 95% CI: 0.66–0.79) as well as in cohort analysis (hazard ratio: 0.78, 95% CI: 0.75–0.82). For comparison purpose, the authors also reported the association between MSDP and offspring type 2 diabetes using the same study designs. The findings showed that there was an increase in risk of type 2 diabetes in children exposed to MSDP in cohort analysis, but this association was attenuated to null in sibling analysis, indicating confounding effects of unmeasured familial factors. Mechanisms through which MSDP influences type 1 diabetes in the offspring are not clear and needs further investigation. One possible explanation is the immunosuppressive effects of nicotine, which may prevent the development of autoimmune diseases, such as type 1 diabetes, by promoting anti-inflammatory processes.

## Maternal smoking and adverse health outcomes

Despite evidence demonstrating a benefit effect of MSDP on offspring type 1 diabetes risk, MSDP should never be considered as intervention to prevent type 1 diabetes in the offspring because of its many other adverse health effects on both children and mothers [1, 10]. An umbrella review showed that MSDP is associated with increased risks of 20 infant-related and seven mother-related health conditions [1]. Most of these association are very likely to be causal and some can have long-term impacts across different phases of life from perinatal to adulthood. MSDP is associated with 3-fold increased risk of sudden infant death syndrome, 2-fold increased risk of asthma, and 1.5-fold increased risk of low birth weight, stillbirth, and obesity in the offspring. Women who are exposed to smoking during pregnancy are estimated to have 2.5-fold increased risk of spontaneous miscarriage in assisted reproduction and ectopic pregnancy. Since MSDP is a modified risk factor, its short-term and long-term detrimental effects are avoidable. This underscores the importance of continuous prevention and intervention efforts to reduce prevalence of MSDP and increase smoking cessation earlier before pregnancy, which would significantly benefit not only mother’s own health but also offspring outcomes.

## Conclusions

The study by Wei et al. [8] provided strong evidence of a lower risk of type 1 diabetes for children exposed to MSDP as compared to unexposed children, by using family-based designs of sibling and cousin comparison analyses. However, this should not preclude the development and implementation of tobacco control policies in pregnant women. Family-based, quasi-experimental designs improve causal inference by reducing unmeasured confounding bias from genetic and early environmental risk factors and is therefore a useful approach in studying the associations between maternal exposures and offspring outcomes. Further research is warranted to understand the mechanisms linking MSDP and type 1 diabetes in the offspring.

## Data Availability

Not applicable.
